# The role of water mass advection in staging of the Southern Ocean *Salpa thompsoni* populations

**DOI:** 10.1038/s41598-023-34231-7

**Published:** 2023-05-01

**Authors:** Natasha Henschke, Boris Espinasse, Charles A. Stock, Xiao Liu, Nicolas Barrier, Evgeny A. Pakhomov

**Affiliations:** 1 Department of Earth, Ocean and Atmospheric Sciences, University of British Columbia, Vancouver, BC Canada; 2 Department of Arctic and Marine Biology, UiT The Arctic University of Norway, Tromsø, Norway; 3 Geophysical Fluid Dynamics Laboratory, NOAA, 201 Forrestal Road, Princeton, NJ 08540 USA; 4 MARBEC, University of Montpellier, CNRS, Ifremer, IRD, Sète, France; 5 Institute for the Oceans and Fisheries, University of British Columbia, Vancouver, BC Canada

**Keywords:** Climate sciences, Ecology, Ocean sciences

## Abstract

*Salpa thompsoni* is an important grazer in the Southern Ocean. Their abundance in the western Antarctic Peninsula is highly variable, varying by up to 5000-fold inter-annually. Here, we use a particle-tracking model to simulate the potential dispersal of salp populations from a source location in the Antarctic Circumpolar Current (ACC) to the Palmer Long Term Ecological Research (PAL LTER) study area. Tracking simulations are run from 1998 to 2015, and compared against both a stationary salp population model simulated at the PAL LTER study area and observations from the PAL LTER program. The tracking simulation was able to recreate closely the long-term trend and the higher abundances at the slope stations. The higher abundances observed at slope stations are likely due to the advection of salp populations from a source location in the ACC, highlighting the significant role of water mass circulation in the distribution and abundance of Southern Ocean salp populations.

## Introduction

The salp, *Salpa thompsoni,* is an important grazer in the Southern Ocean^1^, with a wide distribution spanning from the Subtropical Convergence to the Antarctic continent^2^. Hence, *S. thompsoni* can exist over a broad thermal range (− 1.5 to 7 °C), yet have a preference to warmer waters (2–5 °C)^2^ because cooler temperatures reduce their reproductive fitness^3^. As a result, it is more difficult for *S. thompsoni* populations to complete their lifecycle in cooler, high latitude waters, which explains low and highly variable abundances of *S. thompsoni* close to the Antarctic continent^3–7^.

The western Antarctic Peninsula (WAP) is a productive region with high biomasses of Antarctic krill^8^ supporting numerous top predators, including penguins, seals and whales, as well as commercial fisheries^9,10^. It is one of the fastest warming regions in the world, resulting in reduced sea ice extent and duration^11–13^, and in the past two decades has seen increases in *S. thompsoni* abundance^14,15^. Salps are efficient filter feeders and have a grazing pressure that can regionally exceed the total daily primary production^16^. Hence, their presence can increase competitive interactions with other zooplankton, with regional decreases in *Euphausia superba* (hereafter Antarctic krill) abundance and increases in *S. thompsoni*^14^. In the WAP, abundances of *S. thompsoni* are generally low (climatological mean of ~ 0.1 individuals (ind.) m^−3^) yet can vary inter-annually by a factor of 5000^7^. Large scale atmospheric processes, in addition to biological processes (i.e., mortality, recruitment, survival), were shown to explain the large variability observed in Antarctic krill density and demography^17^. Thus, the large variability in salp density in the WAP may also be driven by these processes that influence sea ice extent and water mass circulation^18^ linking long-term *S. thompsoni* abundances to large atmospheric processes such as the Southern Annular Mode (SAM)^15^. Increasing SAM has been associated with earlier sea ice retreat, later sea ice advance and increased upwelling of warm circumpolar deep water (CDW) in the WAP^18^.

Because *S. thompsoni* reproduction is physiologically limited by cold water^3^, it has been hypothesized that the high biomass years, hereafter “salp years”, are a result of warmer water intrusions onto the shelf^7,19^. These intrusions of warmer water could increase local salp abundance in two ways, either by making the environmental conditions more conducive for the salp reproduction, or by the influx of seeding populations occurring from more optimal source locations. Favourable environmental conditions may not persist throughout the winter affecting the reproductive success of *S. thompsoni*^5^. Hence, the “source population” hypothesis is more plausible with recent modeling efforts in the WAP indicating that immigration of *S. thompsoni* individuals is critical to recreate the observed large variations in abundance^20^. While the study by Groeneveld et al.^20^ is an individual-based local salp model that identified that large salp blooms could not occur without a population boost in early spring, it did not model the likelihood of salp populations being transported from a source location in the Southern Ocean into the WAP. This study builds upon the static approach by Groeneveld et al.^20^ by incorporating the effect of water mass circulation on the dispersal and growth of salp populations.

Particle tracking and ocean models are used to explore the connectivity among oceanic systems and improve, (1) the understanding of the plankton dispersal, (2) the identification of spawning grounds and nurseries^21,22^ and/or (3) high-risk areas for exotic species invasions^23^. In the WAP, annual sampling of *S. thompsoni* have been undertaken in the austral summer (January–February) as part of the Palmer Long Term Ecological Research (PAL LTER) program along the Antarctic Peninsula. In this study, we aim to test the “source population” hypothesis by using the results from the ICHTHYOP particle tracking model^24^ fed with 3D velocity fields combined with a *S. thompsoni* population model^25^ to uncover the physical and ecological processes driving the *S. thompsoni* population distribution and abundance at the PAL LTER study region. Specifically, we use modeled velocities to virtually transport salp populations from a potential source location in the ACC, southeast of the PAL LTER study region. While being transported, the salp population experiences the changes in the water parcel’s temperature and chlorophyll *a* concentration, which influences salp growth, mortality and reproductive rates. Given that simulations were run from 1998 to 2015, this study provides a framework to explore how water mass circulation affects *S. thompsoni* distribution dynamics in the WAP, and in particular to determine how much advection may contribute to the large inter-annual variability observed in *S. thompsoni* populations.

## Results

### Environmental conditions

**Figure 1:**
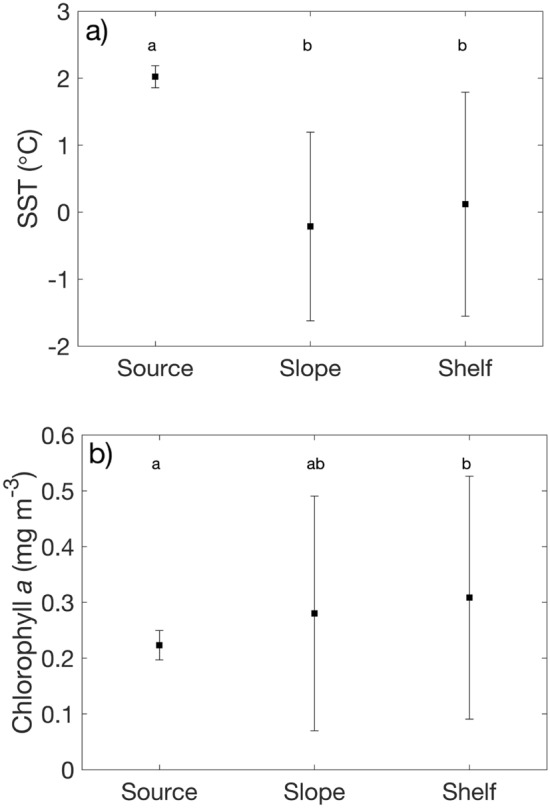
Long-term (1998–2015) mean summer (Dec–Feb) modelled (**a**) SST and (**b**) chlorophyll *a* at the source, slope and shelf regions. Letters denote significant differences at p < 0.05.

Long-term (1998–2015) mean summer (Dec–Feb) modelled sea surface temperature (SST) at the source location was significantly higher than slope and shelf stations in the PAL LTER study region (F_2,162_ = 53, p < 0.001; Fig. 1). Summer (Dec–Feb) modelled chlorophyll *a* concentrations were significantly higher in the shelf stations of the PAL LTER study region compared to the source location (F_2,162_ = 3.39, p = 0.04). There was no significant difference in SST or chlorophyll *a* between shelf and slope stations. While chlorophyll *a* is a necessary input into the *S. thompsoni* model, advection proves to be a more significant driver of variation as seen in the results below.

### Dispersal patterns

**Figure 2:**
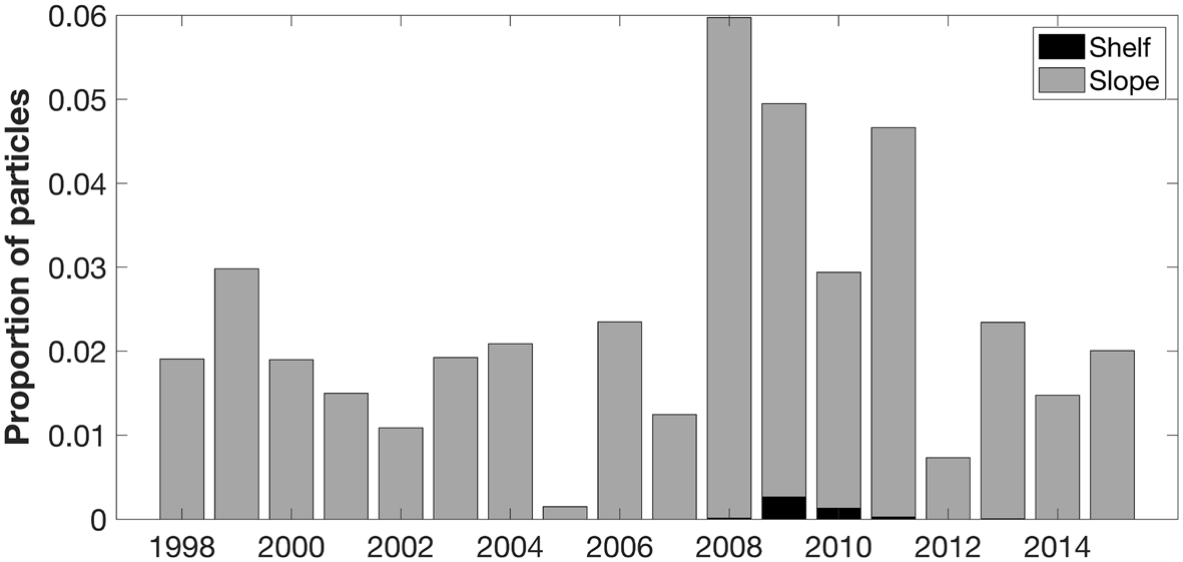
Proportion of particles entering sampling grid during sampling period. Particles only reached the shelf between 2008 to 2011 and in 2013.

The movement of simulated *S. thompsoni* populations were generally consistent with the Antarctic Circumpolar Current, travelling north-east, parallel to the Antarctic Peninsula. The proportion of particles reaching the PAL LTER study region during the sampling period varied each year, ranging from 0.002 (2005) to 0.06 (2008; Fig. 2). The majority (99.5 ± 1.6%) of particles that reached the PAL LTER study region remained on the slope, with particles only reaching the shelf in 5 of the 18 simulated years (2008–2011, 2013).

### PAL LTER observations

**Figure 3:**
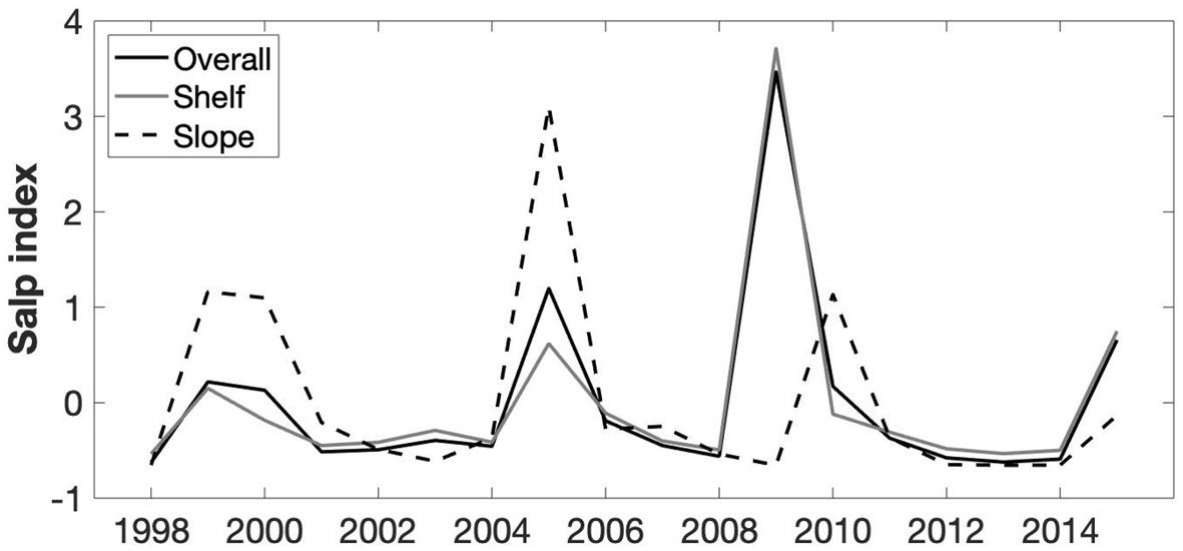
Annual mean salp abundance (salp index) for the PAL LTER study region (overall; solid line), shelf stations (grey line) and slope stations (dashed line).

**Figure 4:**
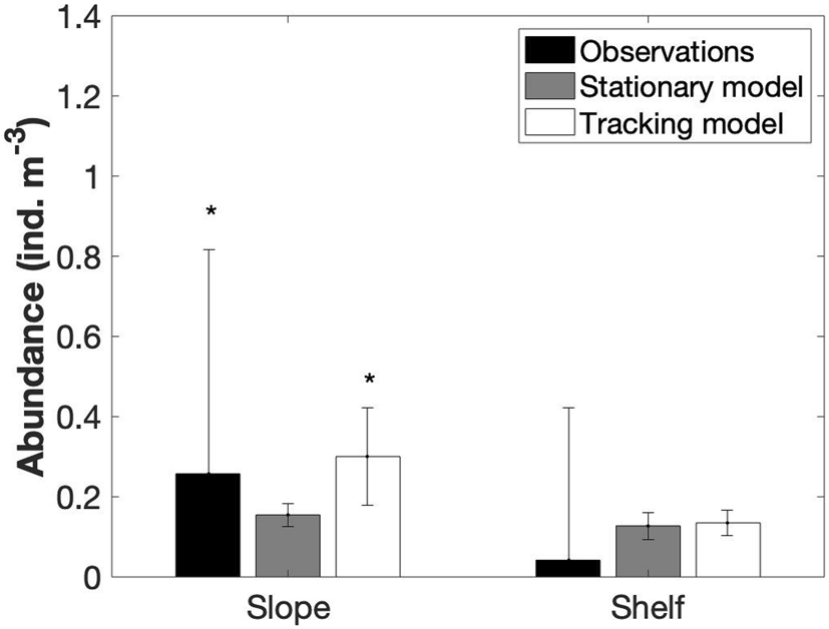
Long-term (1998–2015) mean (± SD) observed and modeled *Salpa thompsoni* abundance in slope and shelf stations of the PAL LTER study region. *significant differences at p < 0.05.

**Figure 5:**
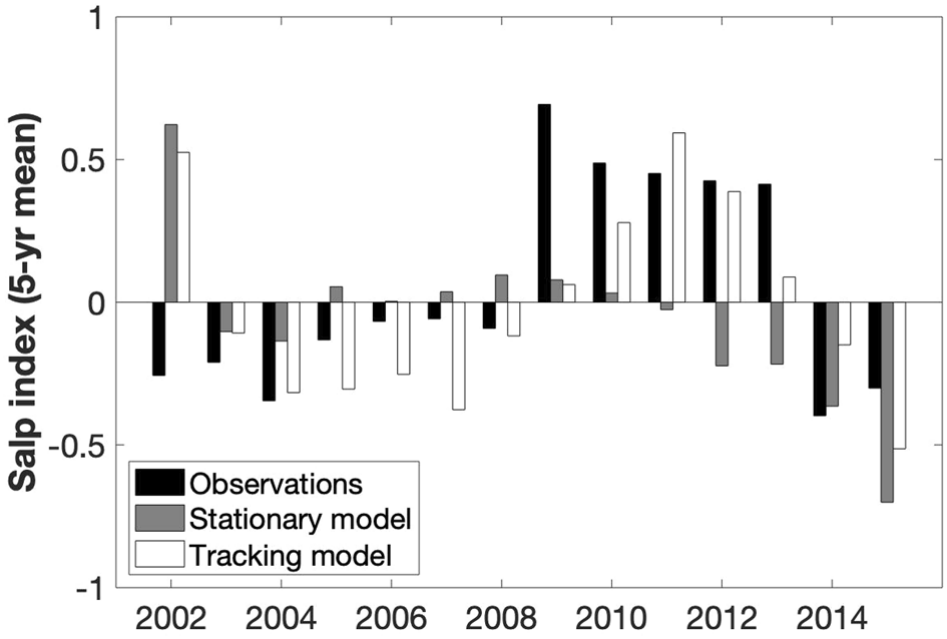
Long-term trends (5-yr mean) in observed and modelled *Salpa thompsoni *abundance at the PAL LTER study region.

The long-term (1998–2015) mean (± SD) *S. thompsoni* abundance observed at the PAL LTER study region is 0.07 ± 0.11 ind. m^−3^. Inter-annual variation in *S. thompsoni* abundance at shelf stations correlated significantly with the inter-annual variation from the whole PAL LTER study region (r = 0.98, p < 0.001), whereas there was no correlation between slope stations and the whole study region (Fig. 3). Long-term mean abundances were significantly higher in slope stations compared to shelf stations (F_1,1128_ = 15, p < 0.001; Fig. 4). The long-term trend indicates a period of low salp abundance from 1998 to 2008 and a period of high abundance from 2009 to 2013 (Fig. 5).

### Simulated salp abundance

The long-term (1998–2015) mean (± SD) *S. thompsoni* abundance simulated by the stationary model (0.14 ± 0.03 ind. m^−3^) and the tracking model (0.19 ± 0.05 ind. m^−3^) were not significantly different from the observations. However, the stationary model could not recreate the higher abundance in slope stations (Fig. 4), or the long-term trend (Fig. 5). In the tracking model, simulated abundances were significantly higher in the slope than in the shelf (F_1,35_ = 30.75, p < 0.001; Fig. 4) and the long-term trend was consistent with the observations (r = 0.75, p = 0.002; Fig. 5), indicating a similar high period from 2009 to 2013. Higher abundances in the tracking model were driven by the number of particles reaching the sampling area (r = 0.78, p < 0.001).

## Discussion

This is the first study to explore the effect of water mass advection on the population density dynamics of *S. thompsoni* populations in the WAP. *S. thompsoni* abundance is highly variable along the WAP, with “salp years” potentially shifting the local ecosystem due to their efficient grazing rates and ability to alter the ecosystem C:N:P stoichiometry^5,26^. Since limited seasonal sampling restricts determination of environmental drivers of inter-annual *S. thompsoni* abundance variability in the WAP, we compared two simulations of a salp population model at the PAL LTER region. A stationary model run precluded the immigration of new individuals while a tracking model through inferred modeled velocities was able to transport *S. thompsoni* populations from a source location in the ACC.

Long-term observations revealed high inter-annual variance in abundance, 20-fold higher abundances in slope stations and a high period of abundance from 2009 to 2013. Salps are historically found in low abundances in the study region, particularly in shelf stations (0.01 ind. m^−3^)^7,15^. Periods of high salp abundance have been related to large scale atmospheric processes, positively correlated with the Southern Annular Mode (SAM) and negatively correlated with the Multivariate El Niño Southern Oscillation Index (MEI)^15^. In the WAP, positive SAM and negative MEI years result in increased northwesterly winds, unfavourable conditions for sea ice and potentially warmer surface waters^15^. Local salp populations in the PAL LTER study region can benefit from warmer surface waters, however, if the increased northwesterly winds alter surface circulation, these conditions could also result in more ACC encroachment into the PAL LTER study region, allowing for increased immigration of new salps^15^.

Both the stationary and tracking models were able to recreate the long-term mean abundance observed for the entire (shelf and slope) PAL LTER study region. This indicates that the model is sufficiently robust to mimic the *S. thompsoni* population in the PAL LTER region by using only temperature and chlorophyll *a* as drivers. However, higher abundances in slope stations were only confirmed when the immigration of new individuals was allowed through the tracking simulations. Subsequently, only the tracking simulations were able to recreate the regional long-term trend of *S. thompsoni* densities. We note, however, that while the 1/4° resolution physical-biogeochemical MOM6-COBALT resolves large-scale spatial and seasonal chlorophyll patterns around the WAP reasonably well, it cannot capture small-scale circulation and frontal features.

The stationary simulation could not recreate the variation in abundance between slope and shelf stations suggesting that thermal and biogeochemical conditions alone were not sufficient to result in consistently higher *S. thompsoni* abundances over the slope. Indeed, SST and chlorophyll *a* concentrations were not significantly different between shelf and slope stations. At the same time, the source location was significantly warmer, and had lower chlorophyll *a* concentration. *S. thompsoni* abundances generally increased with increasing temperature and decreasing chlorophyll *a* concentrations^3,25^, indicating that the source location could be a more optimal habitat for *S. thompsoni* populations to thrive. The observed long-term trend in *S. thompsoni* abundance was highly correlated with the number of particles arriving in the PAL LTER study region, and these particles rarely reached the shelf stations. Thus, if populations at the source location were able to develop more successfully and then were advected onto the slope, this could explain the higher salp abundances observed at the slope stations as well as the large inter-annual variation in the *S. thompsoni* abundance.

The agreement between the number of particles reaching the slope stations, and the higher abundance observed in slope stations highlights the significant role of circulation in Southern Ocean salp populations, and provides a strong support for the “source population” hypothesis. While there is uncertainty surrounding the frequency and magnitude of the advection of *S. thompsoni* populations into the PAL LTER study region, this study postulates that the immigration of new populations is necessary to recreate the observed shelf-slope gradients and long-term trends in salp abundance. This study highlights the critical importance of understanding the circum-Antarctic connectivity of the *S. thompsoni* metapopulation in the Southern Ocean and the significance of regional specifics in salp population developments. Identifying if there are genetic links between areas will be useful in determining how connected the *S. thompsoni* populations are in the Southern Ocean.

In the long-term, as the ACC moves poleward and closer to the shelf break^27–29^, the advection of *S. thompsoni* into the PAL LTER study region is likely to increase in the future. While temperature and chlorophyll *a* are important drivers of salp distribution and abundance, in order to understand the large-scale variations in inter-annual salp abundance, it is necessary to consider the effect of water mass advection and identify the source location of potential seeding populations. A critical reason to increase our understanding of these impacts are to understand the effect that shifting salp populations could have on the food web dynamics in the region^30–32^.

## Methods

### Study site and observations

**Figure 6:**
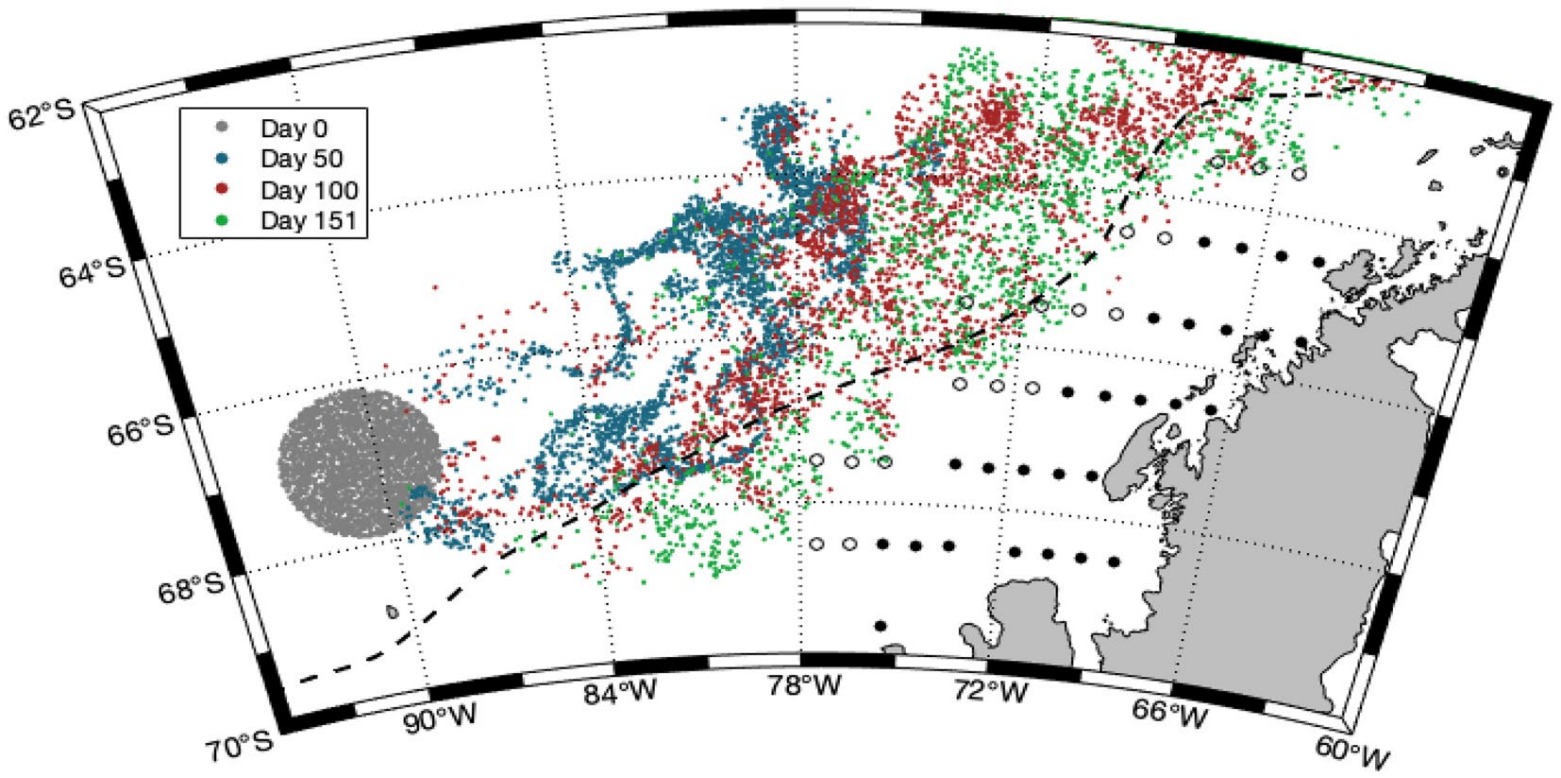
Sampling area indicating the PAL LTER study region and the locations of particles for the 1998 forward simulation. Particle locations during the simulation are represented by the coloured points: the initial release site (i.e. source location; day 0; grey points), day 50 (blue points), day 100 (red points) and the final location (day 151; green). Approximate *Salpa thompsoni* sampling stations in the PAL LTER study region are represented by black (shelf) and white (slope) circles. The southern boundary of the Antarctic Circumpolar Current is indicated by the dashed line. Figure was created using MATLAB version 9.7 (R2019b), Natick, Massachusetts: The MathWorks Inc.; 2019.

The PAL LTER sampling stations are located on the western side of the Antarctic Peninsula. The sampling region extends southwards from Anvers Island (~ 63 °S) to Charcot Island (~ 69° S; Fig. 6). Grid lines are 100 km apart, and extend perpendicular from the coast, extending approximately 200 km offshore to slope waters. Stations along grid lines are spaced approximately 20 km apart and are separated as shelf (0–750 m) or slope (> 750 m). Zooplankton sampling was undertaken annually (1998–2015) during the austral summer (Jan–Feb; Table S1) using a 1 m^2^ (335 μm Nytex mesh) and a 2 m^2^ (700 μm knotless nylon square mesh) Metro net towed obliquely from the surface to 300 m and 120 m respectively. For more detail on the study site and sampling method see Ross et al.^7^. These data are sourced from the PAL LTER data catalog (https://pal.lternet.edu/data).

### Particle tracking

The velocity fields were extracted from the GLORYS12V1 product distributed by the Copernicus platform (marine.copernicus.eu). The GLORYS12V1 product (https://doi.org/10.48670/moi-00024) is the CMEMS global ocean eddy-resolving reanalysis covering the altimetry era 1993–2018^33,34^. We used daily mean files of currents (U, V and W) from the surface to the 1000 m depth, displayed on a standard regular grid at 1/12° (approximately 8 km) and on 35 standard levels. It is based largely on the current real-time global forecasting CMEMS system. The model component is the NEMO platform driven at the surface by ECfMWF ERA-Interim reanalysis. Observations are assimilated by means of a reduced-order Kalman filter. Along track altimeter data (Sea Level Anomaly), satellite Sea Surface Temperature, Sea Ice Concentration and in situ temperature and salinity vertical profiles are jointly assimilated.

Particle trajectories were simulated using ICHTHYOP (www.ichthyop.org), an individual-based model designed to study how physical and biological factors affect plankton dynamics^24^. Daily velocity fields from GLORYS12V1 were used to compute Lagrangian trajectories with the forward Runge–Kutta method (RK4). The computational time step was set to 1800s, ensuring that a particle does not move longer than one grid cell per time step.

A patch consisting of 3000 particles was released into a circular domain with a 200 km diameter at the source location (67° S and 90.5° W; Fig. 6). Horizontal diffusion was implemented according to Peliz et al.^35^ where the Lagrangian horizontal diffusion (K_h_) is defined as:

$${\text{K}}_{{\text{h}}} \, = \,\varepsilon^{{{1}/{3}}} {\text{l}}^{{{4}/{3}}}$$

where ε is the turbulent dissipation rate and l the grid cell size (8 km in GLORYS12v1). The turbulent dissipation rate has been set to 10^–10^ resulting in K_h_ equal to 75 m^2^ s^−1^, which is in accordance with other studies^36,37^. The particles were released the October 1st of each year (1997–2014) and the trajectories were simulated for 151 days until the end of February. Salp diel vertical migration (DVM) was included with particles close to the surface (50 m depth) during the night and distributed deeper in the water column (200 m) during the day, assuming sunrise and sunset time of 6 am and 9 pm, respectively.

The source location and release time (Fig. 6) was defined by backtracking particles from the PAL LTER study region for several years (Fig. S1). Several sources were considered. Most of the particles originated from the surface layer (0–300 m) upstream the ACC while another regular source was located directly offshore the study area, with particles slowly advected onto the shelf from deeper layers. The former source was found to better reproduce observed patterns and was therefore selected. The distribution of particles from the selected source location resulted in a significant portion of particles being transported onto the shelf with their position on the shelf agreeing with locations of *S. thompsoni* documented in the literature^7,15^. An October 1st release date was chosen as it took approximately 3–4 months for particles to reach an ACC source location south of the PAL LTER study region, and because *S. thompsoni* reproduction begins in spring^2^.

### Virtual salp populations

Each particle is assumed to be a 1 m^3^ parcel of water, containing a *S. thompsoni* population. The salp population is simulated by the model developed by Henschke et al.^25^. This is a size-structured *S. thompsoni* population model that incorporates temperature and chlorophyll *a* dependent growth, consumption, reproduction and mortality^25^.

*S. thompsoni* population dynamics are forced with sea surface temperature (SST) and chlorophyll *a* extracted from a ¼° horizontal resolution global ocean simulation from the Modular Ocean Model 6 (MOM6)^38^ that included the Carbon Ocean Biogeochemistry and Lower Trophics (COBALT) plankton ecosystem model^39,40^. COBALT was used as it contains both SST and chlorophyll *a* values as opposed to GLORYS12V1 from where the particle tracking fields were derived which does not contain chlorophyll *a.* Modelled SST and chlorophyll *a* data were used to ensure the most robust coverage in the area, as satellite data from the Southern Ocean are limited due to the region often being obscured by clouds. The COBALT model has proven to be effective in recreating long-term patterns in SST (r = 0.97, p < 0.001) and chlorophyll *a* (r = 0.71, p < 0.01) globally when compared against in situ observations and satellite data (SST and chlorophyll *a*) (Table S2). The COBALT model could also recreate the monthly (r = 0.59, p < 0.04, n = 12) and inter-annual (1996–2007; r = 0.66, p = 0.02, n = 12) trends observed from in situ samples of chlorophyll *a* from the Palmer Station seawater intake (1992–2018). SST trends in COBALT correspond well with large scale monthly SST (r = 0.94, p < 0.001, n = 240) trends from GLORYS12V1 in the WAP.

To compare the effect of advection, two simulations are performed: a stationary simulation and a tracking simulation. Both simulations are run using a 5-year model spin-up prior to commencing to ensure the salp population has reached steady state using the local climatological mean values for SST and chlorophyll *a*. The stationary simulation is run for the sampling period (1998–2015) across grid cells in the PAL LTER study region. The tracking simulations begin at the source location (Fig. 6) where the 5-year spin up is run, ending by October 1. The particles begin within a circle, and after October 1 until the end of the simulation (February 28), as the particles are moved their temperature and chlorophyll *a* concentration trajectory will vary from other particles based on their movement pattern. Each year of the tracking simulation is run separately, so populations will not cumulatively increase throughout the years as advection continues. The *S. thompsoni* life cycle can be completed within a year, hence accumulation is not likely^25^. Inter-annual abundances are calculated based on the mean abundance within the PAL LTER study region from the duration of the sampling period (Table S1). Inter-annual abundances for the tracking simulation are the combined abundances of the local salp populations (stationary simulation) and the influx of additional salps (tracking simulation). The sampling period occurred during January/February (Table S1), and as the tracking simulation was still running until February 28th, this ensured that particles were able to be advected out of the study region as well. Modelled and observed salp abundances were both standardised to an annual mean of 0 and a standard deviation of 1 to create a salp index. A similar method was used to compare jellyfish abundances across diverse metrics^41^. An analysis of variance was used to compare differences in environmental conditions or salp abundances between regions (source, shelf, slope). Pearson correlations were used to compare interannual variation in abundances between regions.

## Supplementary Information

Supplementary Information.

## Data Availability

The zooplankton dataset was obtained from the Palmer LTER data catalog: Palmer Station Antarctica LTER, D. Steinberg, R. Ross, and L. Quetin. 2020. Zooplankton collected aboard Palmer Station LTER annual cruises off the western antarctic peninsula, 1993–2008. ver 4. Environmental Data Initiative. https://doi.org/10.6073/pasta/03e6d72a78bc2512ef5bb327e686f8fa (Accessed 2023-02-09). Additional datasets generated during and/or analysed during the current study are available from the corresponding author on reasonable request.
